# Confirmed Case of Autochthonous Human Babesiosis, Hungary

**DOI:** 10.3201/eid3009.240525

**Published:** 2024-09

**Authors:** Dávid Sipos, Ágnes Kappéter, Barbara Réger, Gabriella Kiss, Nóra Takács, Róbert Farkas, István Kucsera, Zoltán Péterfi

**Affiliations:** University of Pécs, Pécs, Hungary (D. Sipos, Á. Kappéter, B. Réger, G. Kiss, Z. Péterfi);; University of Veterinary Medicine, Budapest, Hungary (N. Takács, R. Farkas);; National Public Health Center, Budapest (I. Kucsera)

**Keywords:** human babesiosis, *Babesia microti*, *Babesia divergens*, *Babesia venatorum*, parasites, vector-borne infections, zoonoses, Hungary

## Abstract

We report a case of autochthonous human babesiosis in Hungary, confirmed by PCR and partial sequencing of the *Babesia* spp. 18S rRNA gene. Babesiosis should be considered during the differential diagnosis of febrile illnesses, and peripheral blood smears to detect *Babesia* spp. should be part of the routine clinical workup.

Since the first description of human babesiosis caused by *Babesia divergens* protozoa in 1956 in the former Yugoslavia, 2 other zoonotic species, *B*. *venatorum* and *B*. *microti*, have been isolated in Europe. Unlike in North America, where most identified human cases have been caused by *B. microti*, the predominent pathogen causing babesiosis in Europe is *B. divergens* (*1*). The rising number of identified human infections in Europe has drawn attention to this emerging tickborne zoonotic disease. In Europe, *B. microti* has been identified in 25 of 71 confirmed human babesiosis cases, of which 11 were autochthonous (*1*). Rodents and insectivores are reservoir hosts for *Babesia* spp. parasites, which are transmitted by widespread *Ixodes ricinus* ticks, well-known vectors of other zoonotic pathogens (*2*). We report a confirmed case of human babesiosis caused by *B. microti* infection in Hungary.

A 64-year-old man who lived in the countryside and worked as a farmer sought care at an emergency department on July 7, 2021, because of fatigue, nausea, vomiting, and a 10-kg weight loss during the past 2 months. His body temperature reached 38.9°C. He was unaware of having any chronic illnesses or tick infestations and did not have a blood transfusion or indicate a travel history outside of Hungary. Routine laboratory tests confirmed slightly elevated bilirubin (2.13 mg/dL), lactate dehydrogenase (760 U/L), alkaline phosphatase (170 U/L), gamma-glutamyl transferase (207 U/L), blood urea nitrogen (36.96 mg/dL), and creatinine (1.27 mg/dL) levels. He also had hyponatremia (120 mEq/L), prominent elevation of ultrasensitive C-reactive protein (218.8 mg/L), and new-onset diabetes, as well as slight anemia (hematocrit 37.9%) and an elevated procalcitonin level (1.94 ng/mL). A complete blood count examined by using an automated hematology analyzer (Sysmex, https://www.sysmex.com) showed elevated monocyte levels (25%) and thrombocytopenia (78 × 10^9^ platelets/L). We examined peripheral blood smears by using automated and light microscopy, which confirmed intraerythrocytic ring forms with central vacuoles, some intraerythrocytic tetrades, and extraerythrocytic forms, suggesting babesiosis rather than malaria (Figure 1). Parasites infected 4.5% of erythrocytes. Because of microscopic findings and laboratory results, hospital staff tested blood haptoglobin level, which was 0.0 mg/dL, confirming suspected hemolysis. Other symptoms appeared during hospitalization, including left subcostal pain, decreased exercise tolerance, constipation, shivering, and new-onset torpidity; blurred vison occurred a few days after admission. Although malaria is not endemic in Hungary, we performed serologic tests for *Plasmodium* spp., which had negative results.

**Figure 1:**
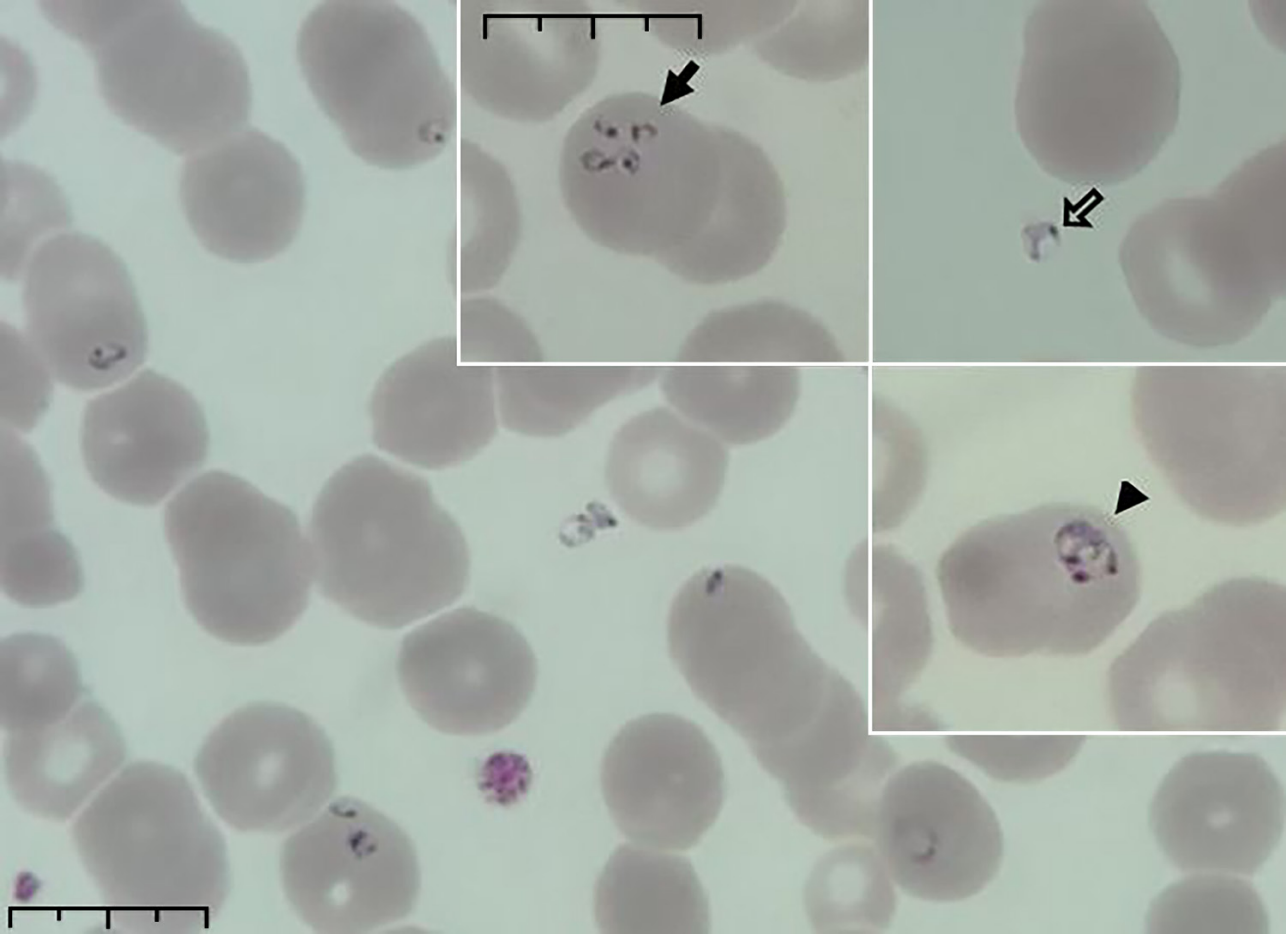
Peripheral blood smear from patient who had a confirmed case of autochthonous human babesiosis, Hungary. Smear shows erythrocytes infected with *Babesia* sp.; smear was stained with May-Grünwald Giemsa stain and examined by using light microscopy. In inset images, solid arrow indicates cells infected with multiple merozoites, open arrow indicates extracellular parasites, and arrowhead indicates vacuolated ring forms (trophozoites). Scale bars indicates 10 μm.

We performed PCR with primers BJ1 and BN2 to amplify a 459-bp fragment of the 18S rRNA gene of *Babesia* sp (*3*). at the University of Veterinary Medicine, Budapest; the fragment was sequenced at the University of Szeged, Szeged, Hungary. We deposited the sequence in GenBank (accession no. OP143843.1). Nucleotide BLAST (https://blast.ncbi.nlm.nih.gov) analysis of the sequence showed 100% homology to *B. microti* detected in *I. ricinus* ticks and human blood (*4*). Although the sequence likely represents *B. microti*, further sequencing was not possible, and another closely related *Babesia* species cannot be excluded. We constructed a phylogenetic tree to compare the sequence with other *Babesia* spp. sequences found in GenBank (Figure 2).

**Figure 2:**
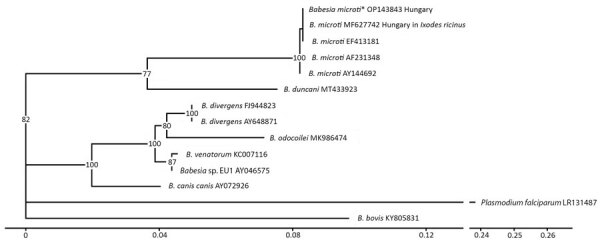
Phylogenetic analysis of *Babesia* spp. in confirmed case of autochthonous human babesiosis, Hungary. Asterisk indicates *B. microti* isolated from the patient in this case study. Phylogenetic tree was constructed by using Ggtree (*5*) according to multiple sequence alignments created by using MAFFT software (*6*). Best substitution model (3-parameter model, TPM2u) was selected by using functions of the phangorn version 2.11.1 R package (*7*) according to the Bayesian information criterion. Neighbor-joining tree was optimized by using the maximum-likelihood method. Bootstrap values were produced by 100 iterations and are indicated at branches. All data processing and plotting were performed in R version 4.4.1 (The R Project for Statistical Computing, https://www.r-project.org). GenBank accession numbers are indicated after the species name. Scale bar indicates nucleotide substitutions per site.

Because of the advanced age of the patient and the clinical picture, we administered atovaquone/proguanil and azithromycin for 2 weeks (atovaquone is available in Hungary as an antimalaria drug). The patient became afebrile, and his condition improved. During follow-up examinations, fatigue and blurred vision gradually disappeared, and laboratory results improved. On the eighth day after treatment ended, we were unable to see any parasites through microscopic examination of blood. By the ninth week after treatment, lactate dehydrogenase, haptoglobin, and hemoglobin levels had normalized, and the patient fully recovered.

Although most human babesiosis cases have been imported in Europe, an increasing number of autochthonous *Babesia* spp. infections have been reported, possibly from a greater chance of tick contact because of human behavior changes (e.g., seeking outdoor activities) and a surge in vector population because of climate change. Furthermore, the number of immunocompromised hosts who have a more severe disease course and seek medical care is increasing as well.

In conclusion, although the 2 zoonotic species *B. divergens* and *B. microti* and their *I. ricinus* tick vector can be found in Hungary (*8*), imported or autochthonous human babesiosis cases had not been reported in this country. Babesiosis is not an endemic disease in Hungary; thus, clinicians rarely suspect this disease, despite the typical symptoms. Seroepidemiologic findings confirm the possibility of *Babesia* spp. transmission to humans in Europe. The increasing number of reported cases indicates that babesiosis should be considered in the differential diagnosis of patients manifesting fever in Europe. Furthermore, peripheral blood smears to detect this parasite should be a routine part of the workup for febrile illnesses, especially when disease-typical laboratory findings are present.
